# Preparation of surgical meshes using self-regulating technology based on reaction-diffusion processes

**DOI:** 10.1007/s11517-024-03141-9

**Published:** 2024-06-05

**Authors:** Péter Polyák, Katalin Fodorné Vadász, Dóra Tátraaljai, Judit E. Puskas

**Affiliations:** 1 Laboratory of Plastics and Rubber Technology, Department of Physical Chemistry and Materials Science, Budapest University of Technology and Economics, Műegyetem rkp. 3., Budapest, 1111 Hungary; 2 Department of Food, Agricultural and Biological Engineering, The Ohio State University, 1680 Madison Avenue, Wooster, 44691 OH USA; 3 Institute of Materials and Environmental Chemistry, Research Centre for Natural Sciences, Magyar Tudósok Körútja 2., Budapest, H-1117 Hungary

**Keywords:** Surgical mesh, Reaction-diffusion, Self-regulation, Self-assembly, SIBS

## Abstract

**Abstract:**

While reaction-diffusion processes are utilized in multiple scientific fields, these phenomena have seen limited practical application in the polymer industry. Although self-regulating processes driven by parallel reaction and diffusion can lead to patterned structures, most polymeric products with repeating subunits are still prepared by methods that require complex and expensive instrumentation. A notable, high-added-value example is surgical mesh, which is often manufactured by weaving or knitting. In our present work, we demonstrate how the polymer and the biomedical industry can benefit from the pattern-forming capabilities of reaction-diffusion. We would like to propose a self-regulating method that facilitates the creation of surgical meshes from biocompatible polymers. Since the control of the process assumes a thorough understanding of the underlying phenomena, the theoretical background, as well as a mathematical model that can accurately describe the empirical data, is also introduced and explained. Our method offers the benefits of conventional techniques while introducing additional advantages not attainable with them. Most importantly, the method proposed in this paper enables the rapid creation of meshes with an average pore size that can be adjusted easily and tailored to fit the intended area of application.

**Graphical abstract:**

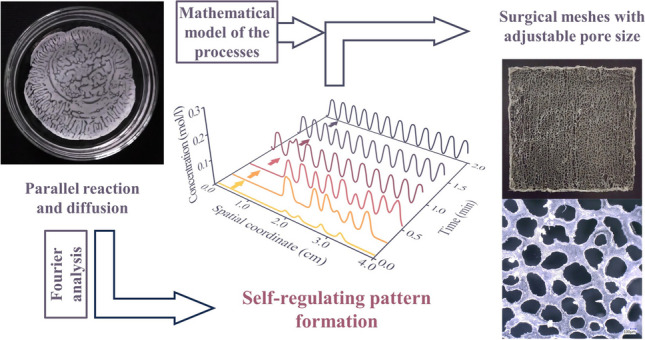

**Supplementary Information:**

The online version contains supplementary material available at 10.1007/s11517-024-03141-9.

## Introduction

**Fig. 1:**
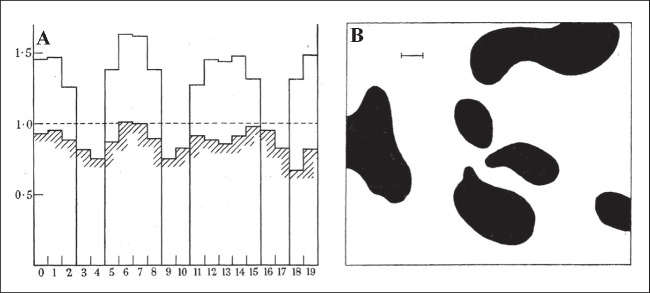
Formation of reaction-diffusion patterns in one-dimension (**a**) and two-dimensions (**b**), as presented in Turing’s original article [2]. In his paper, these diagrams lack the names of axes. Each axis represents spatial coordinates except the vertical axis of (**a**), which represents concentration

The self-regulating biochemical processes that take place in living organisms are of extreme complexity. Although the exact quantitative and qualitative analysis of all the reactions that occur even in a single cell is nearly impossible, there is a long history of attempts that targeted the description and characterization of the phenomena that shape living organisms [1]. The first article that had a profound influence on the physicochemical theories of self-organization was written by Alan Turing in 1952 [2]. Turing was a true modern polyhistor: although he is mainly renowned for his contribution to breaking the Enigma code and for being a pioneer in computer science and artificial intelligence, he also laid the foundations of mathematical biology [2]. In his 1952 article, ‘The chemical basis of morphogenesis’, Turing hypothesized that pattern formation in living organisms could be explained by considering only two parallel phenomena: reaction and diffusion [2]. Despite the availability of high-performance computers, Turing correctly predicted what the patterns formed by parallel reaction and diffusion would look like; see Fig. 1 [2].

Although Turing’s work in this field is now regarded as groundbreaking, his contemporaries did not receive his ingenious ideas well. Waddington [3], one of the most prominent developmental biologists of the post-war era, explicitly opposed the inclusion of Turing’s concept in scientific discourse. As a result, reaction-diffusion models were largely overlooked for the next two decades. In 1972, Gierer and Meinhard practically re-discovered the idea of reaction-diffusion models and proposed a mathematical method that can predict the formation of patterns observable in nature [4]. Gierer and Meinhardt’s second pivotal contribution to this field was the empirical validation of reaction-diffusion models. Their model organism was a freshwater polyp hydra (*Hydra attenuata*) [5–9]. Later, Yamaguchi carried out similar experiments with zebrafish (*Danio rerio*) and proved that the Gierer-Meinhardt model can be used to describe the formation of patterns on vertebrates as well [10].

Although the Gierer-Meinhardt model and its derivatives are widely accepted among biologists, the importance of reaction-diffusion phenomena is certainly not limited to biology. In the past few decades, several other models have emerged that can describe a variety of processes not related to biology or biochemistry in any way. The list of notable examples includes the Brusselator model [11], FitzHugh-Nagumo model [12], and, most importantly, the Gray-Scott model [13]. Since its first appearance in 1983, the Gray-Scott model has gained popularity across a surprisingly large number of scientific fields. Chemists mainly use it to optimize batch reactors [14, 15] and plug-flow reactors [16]. Bhattacharjee reported that the model can also provide a basis for the numerical analysis of hydrodynamics of reactive mixtures [17]. Recently, the model was successfully applied to simulate the structure of carbide composites [18, 19]. Besides being able to simulate or predict, the Gray-Scott model can also inspire: Hankins argued that biomimetic design for composites could enhance their properties and generated bio-inspired patterns with the Gray-Scott model [20]. While the articles cited above discuss comparatively simple processes with only a few components, the model was proven to be able to describe reactions in extremely complex environments. A notable example is its application in neurosciences [21, 22], or for the purpose of building gene networks [23].

The number and diversity of the articles cited above highlight the immense potential offered by reaction-diffusion models. Interestingly, in the field of polymer science, their impact is still marginal. Although many manufacturing techniques could be based on the pattern-forming capabilities of reaction-diffusion phenomena, none of the processing methods proposed in the literature or applied by the industry leverage this potential. There are numerous polymeric products composed of regular or semi-regular patterns that are manufactured by conventional processing technologies. Notable examples are surgical meshes, which are widely used in regenerative medicine [24–26]. Products that have received regulatory approval and are currently in use are often manufactured by knitting [27, 28] or weaving [29, 30]. Both techniques require complex instrumentation that is also rather expensive to operate. Therefore, new methods that can output nonwoven fabrics have also emerged. The most relevant examples are electrospinning [31] and 3D printing [32]. Although these techniques are promising, they have not yet proliferated in the market and are, in most cases, still in the development stage [31, 32].

Consequently, the biomedical industry could certainly benefit from a new method that does not require expensive instrumentation and can also be upscaled easily and rapidly to satisfy the demand of the market. We would like to propose self-regulating reaction-diffusion processes that were shown to be able to produce patterns very similar to that of surgical meshes already used in regenerative medicine [33]. Besides the technique, the polymer is also to be selected. We aim to demonstrate the potential of our method using poly(styrene-*b*-isobutylene-*b*-styrene), abbreviated as SIBS. SIBS is an elastic block copolymer that consists of a rubbery phase containing polybutadiene segments and a glassy phase containing polystyrene segments [34].

This polymer has been selected for multiple reasons. Firstly, SIBS is a perfect candidate for the role of an implant material. It is inert, biocompatible, does not trigger allergic or hypersensitivity reactions, and is also easily sterilizable [34–36]. Secondly, SIBS has been proven to be able to function as a drug carrier matrix, a quality often expected of modern implants [34, 36]. Furthermore, its mechanical properties are well-suited for long-term use as a surgical mesh. Puskas extensively studied the mechanical characteristics of SIBS and reported modulus, tensile strength, and elongation at break values [37, 38] that surpass those specified as a requirement for surgical meshes [39]. Due to the continuous, repetitive movement of the human body, the implant must also be able to withstand cyclic mechanical load. El Fray studied the dynamic fatigue properties of SIBS thoroughly and concluded that this polymer meets these requirements as well [35].

Practical considerations also influenced our choice. The first one is price: if the technology arrives at the stage of scaling up, the price of the polymer becomes a critical factor. Manufacturers, such as Kaneka [40], typically offer this polymer at 6-8 $/kg, making it affordable even if large volumes are to be purchased. The last and maybe the most important factor that highlights the suitability of SIBS for the role of an implant is its current application as a coronary stent. The Boston Scientific Corporation introduced its SIBS-based coronary stent into the market as early as 2002 under the trade name TAXUS. Since then, the stent has been implanted into millions of patients [41–43]. By using our method presented in this paper, the self-regulating production of surgical meshes also becomes possible.

## Experimental

### Experiments that target the analysis of pattern formation

SIBS (courtesy of Kaneka) was supplied under the trade name SIBStar 073T. According to the datasheet of the product, the polystyrene content of this grade is 30%, while the average molecular weight is below 100,000 Da. First, the polymer was dissolved in chloroform (Molar Chemicals Ltd.) at the boiling point of the solvent (approximately 62 °C); the dissolution lasted an hour under constant reflux and stirring at 300 RPM. Solutions of two different concentrations were prepared: 4 and 5 m/m% with respect to the polymer. Prior to the preparation of reaction-diffusion patterns, the solutions were cooled down to room temperature, as increased temperatures could alter the process of pattern formation drastically. Therefore, reaction-diffusion patterns were created at room temperature by using the method implemented as follows. A Petri dish of 5 cm inner diameter was filled with the precipitator medium; for this purpose, methanol, ethanol, n-propanol, i-propanol, and tert-butanol were used. All alcohols were supplied by Molar Chemicals Ltd. except tert-butanol, which was purchased from Fluka Chemicals.

Then, 300 μl of the solution of the polymer was dispensed from a 1000 μl pipette tip at the very bottom of the precipitator medium, i.e., the pipette tip was lowered to touch the center of the circular bottom of the Petri dish. In this position, the solution of the polymer was dispensed in approximately 3 s. Due to the high density of the solution (~1.4 g/ml), it spread out under the less dense precipitator medium (~0.8 g/ml, depending on the aliphatic chain of the alcohol) without visible precipitation in the first few seconds. Then, the precipitation and the formation of patterns started, which was monitored by a camera placed above the Petri dish. In order to maximize contrast, black paper was laid under the Petri dish and served as a background of the white polymer. In addition to the video that recorded the process of pattern formation, a static photo was also taken one hour after the start of the experiment. As the process reaches its final state in one hour, photos taken at later times show the exact same pattern. Therefore, a photo taken one hour after the start of the experiment can be considered to represent the ‘after infinite amount of time’ state of the pattern formation and was used as a basis for comparison (i.e., as a control).

### Experiments that target the preparation of surgical meshes

Surgical meshes were prepared by a method similar to that described in Section 2.1, with the exception of using an additive. The formation of evenly distributed holes in the mesh was aided by polyethylene glycol (PEG). In our experiments, PEG with an average molecular weight of 6000 g/mol was used; the product was supplied by Sigma-Aldrich. The preparation of the solution that contained this additive was carried out as described in Section 2.1, except for the addition of PEG6000 to the mixture prior to the start of the reflux. The best results were achieved with a solution that contained 94 m/m% chloroform, 4 m/m% SIBS, and 2 m/m% PEG6000. Since surgical meshes that are currently available in the market usually have rectangular shapes, these samples were not created in a circular Petri dish. Instead, we let the solution of the polymers spread on rectangular glassware. Once the pattern formation finished, the sample was immersed in an aqueous bath, which made the removal of the polymer from the surface of the glass significantly easier.

## Results and discussion

The results are discussed in multiple sections. First, the process of pattern formation will be presented. In this section, the time and spatial coordinate dependence of the patterns will also be thoroughly characterized. Next, we will propose a mathematical model for the process that explains the regularity observed in the pattered samples. Lastly, we will demonstrate how the results of the pattern analysis, as well as the conclusions of the modeling, can be used to develop a method that enables the self-regulating formation of surgical meshes.

### Analysis of pattern formation

**Fig. 2:**
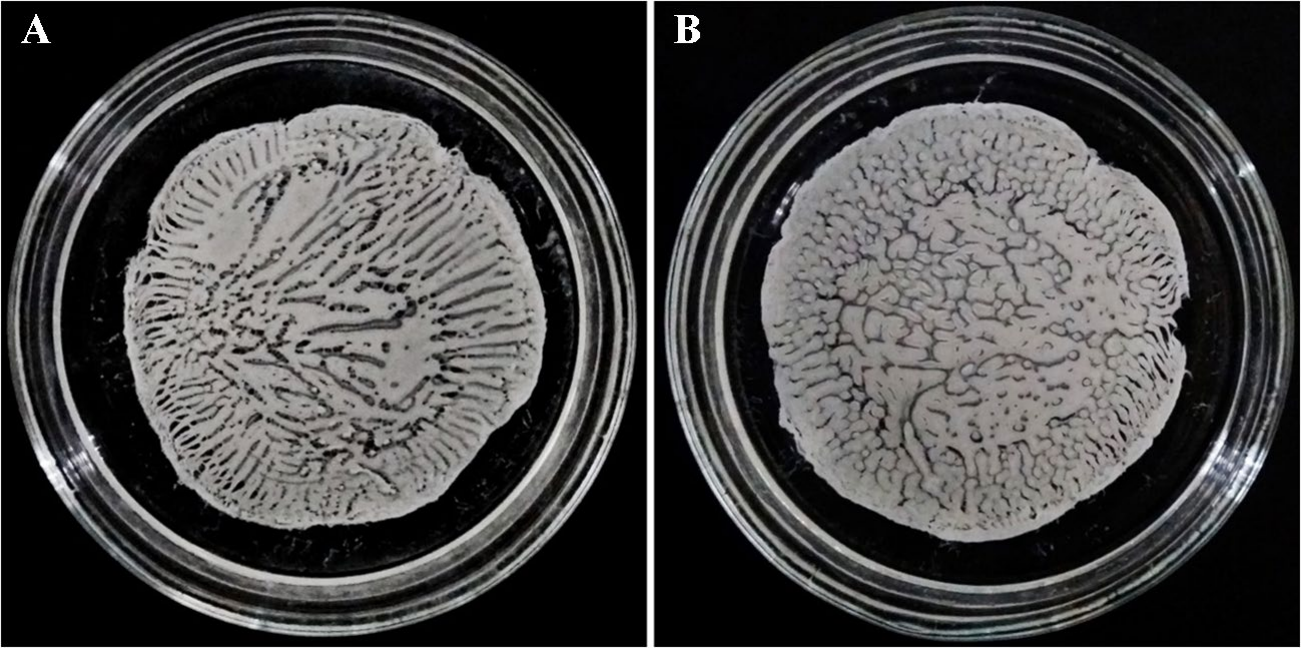
Photo of patterns created from a 4 m/m% solution (**a**), and a 5 m/m% solution (**b**). The precipitator medium was isopropanol in both cases. In spite of the similar initial conditions, the final patterns are completely different

The patterns obtained as a result of the experiments described in Section 2.1 are demonstrated in Fig. 2. The reaction-diffusion patterns observed in this figure are the result of two parallel processes. In these experiments, the reaction is the precipitation of the polymer, whereas the diffusion is that of the macromolecules that have not precipitated yet. Although the technology is rather robust and patterns similar to those shown in Fig. 2 can be created easily, the geometrical characteristics of the final pattern are very sensitive to environmental factors, including contaminations in the precipitator medium or on the surface of the Petri dish. In general, the same pattern cannot be created twice: repeated experiments yield similar, but not identical patterns. While contaminations or impurities cannot be controlled directly, the parameters of the polymer solution and the characteristics of the precipitator medium can. A comparison of Fig. 2a and b reveals that increasing the polymer concentration by 1 m/m% shifts the appearance of the sample from a stripe-dominated pattern to a dot-dominated pattern.

The photos shown in Fig. 2 represent the final (static) stage of pattern formation. Besides static images, videos of the process were also recorded. The video accessible as supplementary file SI1 demonstrates the time-dependence of two patterns. Both experiments were carried out with the same parameters: 4 m/m% polymer solution precipitated in isopropanol. Accordingly, the precipitation starts at the same time and completes at the same time. Likewise, the precipitation starts near the perimeter of the Petri dish and gradually progresses towards its center. Despite the parallel nature of the measurements, different patterns were formed. This difference highlights again that the process is very sensitive, even to minor perturbations. Regarding Fig. 2, we also would like to point out that the process of pattern formation can be disturbed by several factors. These disturbances must be eliminated in order to obtain patterns that can be a basis for subsequent quantitative analysis. The most notable examples are as follows. The Petri dish must be horizontal; otherwise, the unevenly distributed polymer solution will lead to position-dependent spatial frequency. Further, macromolecules are to be precipitated from a completely homogeneous solution because partially dissolved polymer causes irregularities in pattern formation. The importance of working with very pure solvents is also worth emphasizing: contaminations in the polymer solution and/or the precipitator medium can lead to patterns of unpredictable and position-dependent spatial frequency.

**Fig. 3:**
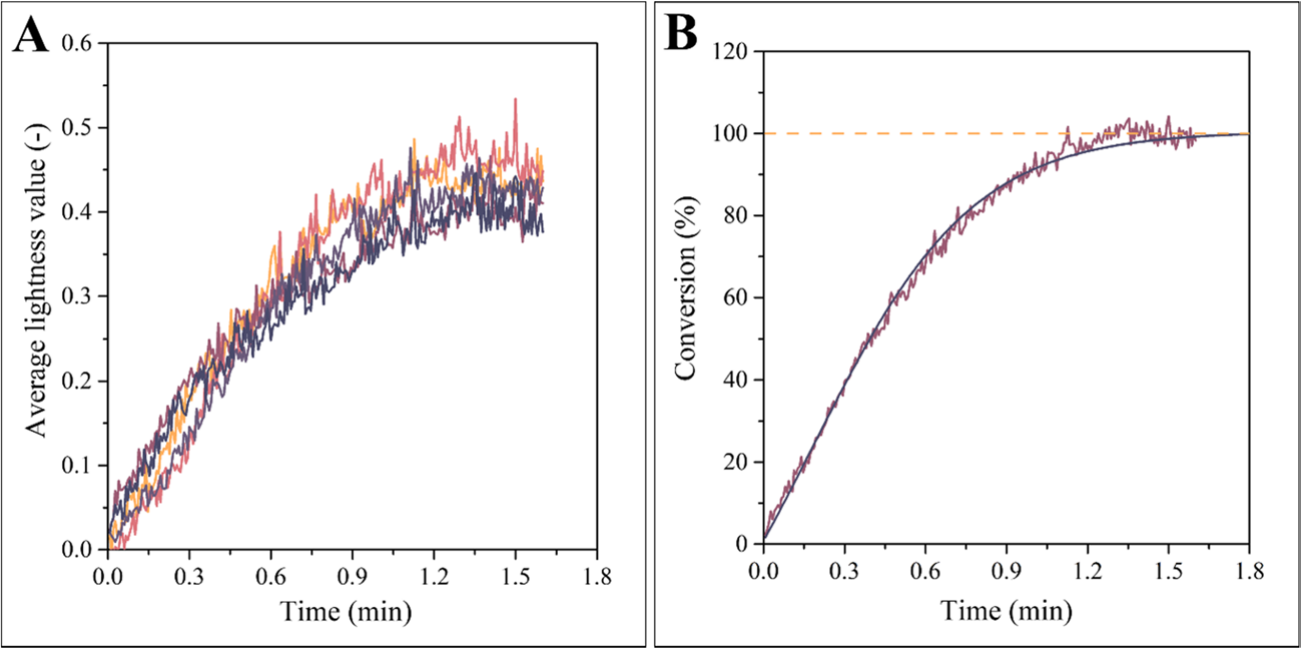
Results of individual measurements (**a**), and the normalized average curve (**b**), which highlights the effectiveness of averaging as a means of improving the signal-to-noise ratio. Note that the conversion converges to 100% with saturation-like kinetics

An additional advantage of recording a video of the precipitation is that in this way, the kinetics of the process can also be analyzed. As a first step, we converted the video to a sequence of images; then, the images were converted from the red-green-blue (RGB) color space to hue-saturation-value (HSV) color space. In HSV color space, the third parameter (value) correlates positively with the lightness of the investigated pixel. The more polymer is precipitated at the investigated point, the brighter the pixel, i.e., the larger the lightness value. Accordingly, the state of the process can be monitored by plotting the lightness values against time. We have averaged the lightness value of all pixels where the polymer was expected to appear (i.e., in the Petri dish), and plotted the averages. Figure 3a demonstrates this plot for five parallel measurements.

Due to the noise superimposed on the raw data, the tendency is difficult to discern. Therefore, the curves were averaged, which reduced the noise significantly. The averaged curve converges to a constant value; this value can be attributed to 100% conversion. The averaged values are to be multiplied by the ratio of 100% and the lightness value the averaged curve converges to. The time-conversion diagram normalized in this way is presented in Fig. 3b. The process follows saturation-like kinetics: at the beginning, the rate of precipitation is at its maximum. Then it decelerates, and the rate gradually converges to zero.

In addition to the time dependence of the process, the spatial coordinate dependence of the patterns was also analyzed. Even though parallel experiments lead to differently shaped patterns, a very important parameter of patterns yielded by parallel experiments does not depend on stochastic factors. This parameter is the spatial frequency, which, in this case, can be defined as the number of stripes (Fig. 2a) or dots (Fig. 2b) per unit distance. Spatial frequency can be calculated by Fourier transformation. Although in many engineering fields Fourier transformation is applied to signals to convert them from the time domain to the (complex) frequency domain, this mathematical apparatus can also facilitate the conversion of spatial functions from real space to reciprocal space.

**Fig. 4:**
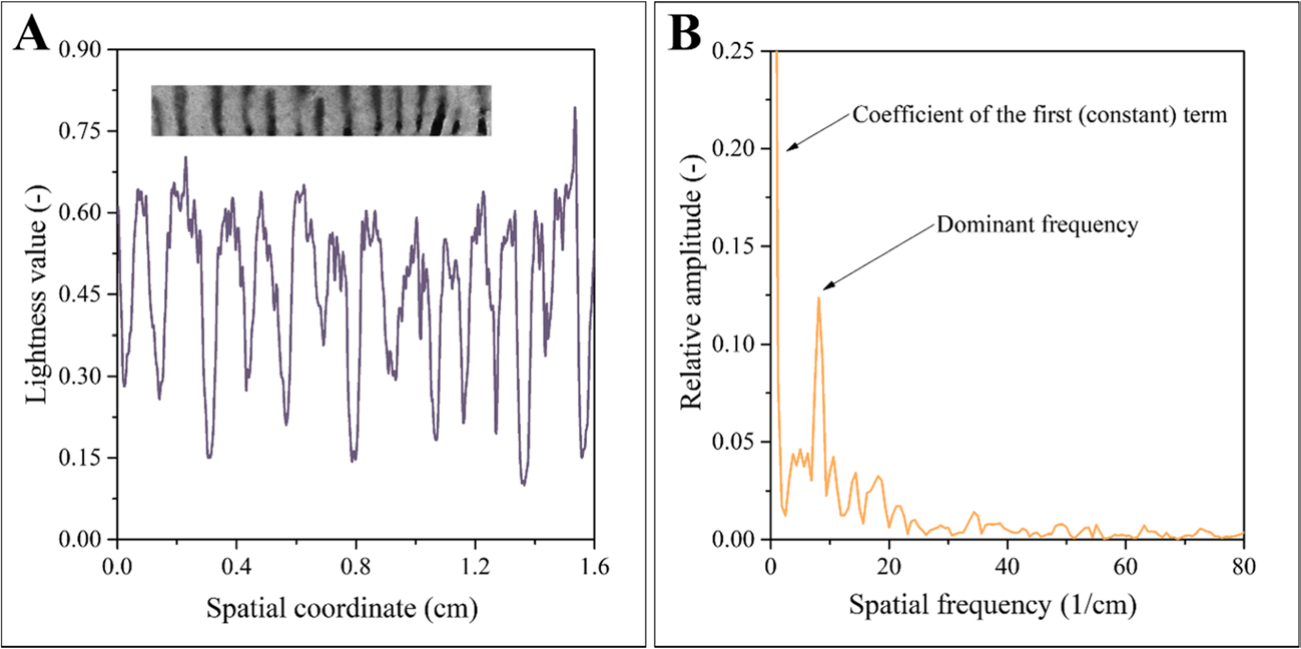
Pattern as a function plotted in real space (**a**), and in reciprocal space (**b**). Only one dominant peak can be observed on the spectrum, i.e., the pattern can be characterized by one spatial frequency

In our study, we aim to use the Fourier transformation as a sophisticated mathematical tool to calculate the frequencies of periodic shapes, such as strips or dots. Technically, Fourier transformation could be performed directly on photos depicted in Fig 2. However, this step would yield two-dimensional spectra, which are challenging to interpret and process further. Consequently, we opted for a different approach. Instead of transforming the entire image, we have extracted small portions of the pattern, such as the one shown in Fig. 4a. This sampling was done in a representative manner, i.e., samples were taken from uniformly distributed locations.

At this point, attention must be drawn to the importance of representative sampling: the results will be accurate and reliable only if all regions of these patterns (see Fig. 2) are analyzed simultaneously. We have found that the computational method is robust and is not particularly prone to the presence of noise. Therefore, noisy regions of the pattern can also be sampled. The calculation will yield the targeted frequency; only the signal-to-noise ratio will be somewhat smaller. The lightness value - spatial coordinate function of a sample obtained in this way is also shown in Fig. 4a. Then, this function was transformed into the reciprocal space by one-dimensional Fourier transformation; see Fig. 4b.

**Fig. 5:**
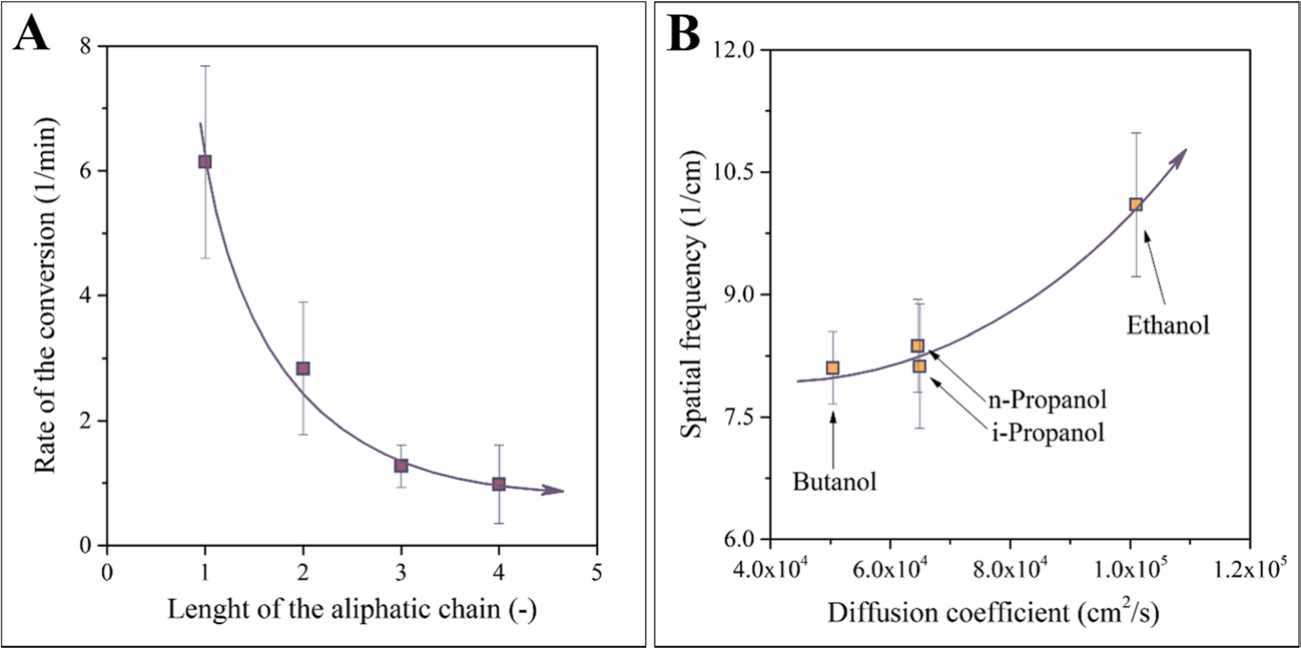
Results of the analysis of kinetics (**a**) and results of the measurement of dominant spatial frequency (**b**). The former offers a visual proof of the observation that pattern formation can be accelerated by using short-chain alcohols as a precipitator medium, whereas the latter highlights that the geometrical characteristics of the patterns can be adjusted by selecting solvents of different diffusion coefficients

By using the methods introduced and explained above, we have analyzed the time and spatial coordinate dependence of these patterns thoroughly. The results are compiled in Fig. 5.

A convenient way to quantize the rate of the conversion graphically represented in Fig. 3b is the calculation of the slope of the regression curve at the beginning of the process. The value obtained in this way is plotted on the vertical axis of Fig. 5a. As an independent variable, the length of the aliphatic chain in the alcohol was used, i.e., the scale ranges from methanol to butanol. The sequence of points outlines a clear tendency: the longer the aliphatic chain, the slower the precipitation. This tendency is due to the chemical potential of SIBS across the homologous series of aliphatic alcohols. The chemical potential of the polymer is the largest in methanol. Accordingly, the precipitation occurs very rapidly here. As the length of the aliphatic chain increases, the chemical potential of the polymer in the alcohol decreases, which also means that the thermodynamic driving force of the material transport becomes reduced. Thus, there is a negative correlation between the rate of process and the length of the aliphatic chain, as proven empirically by Fig 5a.

Figure 5b is dedicated to the second measured parameter, i.e., the spatial frequency of the patterns. Rather than the length of the aliphatic chain, the diffusion coefficient of the alcohol was selected here as an independent variable. Experiments carried out in methanol are omitted because the rapid precipitation leads to patterns that are challenging to analyze accurately. Instead, both n-propanol and i-propanol are represented in this diagram. The tendency outlined by the empirical data reveals that the faster the diffusion of the alcohol, the larger the spatial frequency of the pattern will be. In practice, this means that the number of characteristic geometrical elements (such as stripes or dots) per unit length can be increased by selecting a precipitator medium with a shorter aliphatic chain. The positive correlation demonstrated by Fig 5b also enables the control of the spatial frequency of the pattern.

### Modeling of the process

The first model that was discussed in the introduction was proposed by Gierer and Meinhardt. Although this approach can provide a surprisingly accurate mathematical representation of the reactions that take place in living organisms, we have found that the activator-inhibitor approach the Gierer-Meinhardt model relies on does not comply with the process we are investigating here. In contrast, the Gray-Scott model assumes consecutive reactions, which aligns well with the gradual precipitation of the polymer. Therefore, we opted for the application of the Gray-Scott model, which is generally expressed in the following form:

1 $$\frac{\partial A}{\partial t}={D}_A\bullet {\nabla}^2A-A\bullet {B}^2+f\bullet \left(1-A\right)$$

2 $$\frac{\partial B}{\partial t}={D}_B\bullet {\nabla}^2B+A\bullet {B}^2-B\bullet \left(f+k\right)$$

**Fig. 6:**
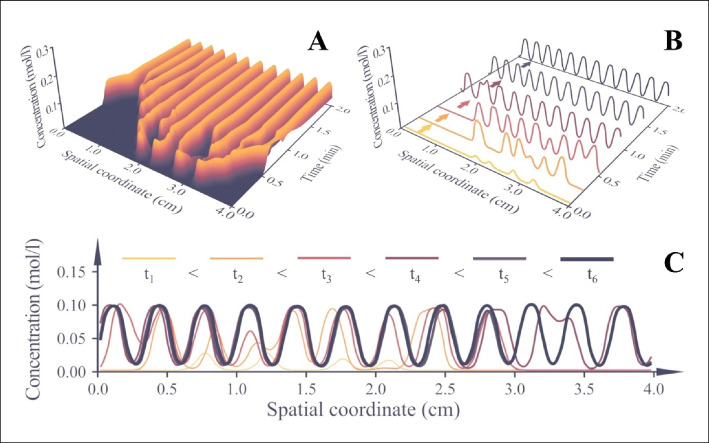
Results of modeling the process in one dimension. Time-dependent concentration profile plotted as a surface function (**a**), and the convergence of concentration profile to a perfectly regular, sinusoidal pattern (**b**). The lowermost figure (**c**) demonstrates the formation of the sinusoidal pattern as a conventional, two-dimensional diagram

In our experimental arrangement, *A* and *B* represent the amount of dissolved polymer and the polymer being precipitated, respectively. Parameter *f* stands for a rate of material transport: the translation of alcohol molecules into the solution of the polymer that continuously increases the number of macromolecules that will be precipitated. Parameter *k* marks the rate of precipitation, whereas *D*_*A*_ and *D*_*B*_ are the diffusion coefficients. The nabla operator represents the partial derivative with respect to spatial coordinates; the second independent variable (time) is denoted by *t*. First, the differential equation system was solved in one dimension. For this purpose, our research team wrote a purpose-specific software in MATLAB. The time and spatial coordinate dependence of the solution is shown in Fig. 6 and is also illustrated by the video available as supplementary file SI2.

The solution takes the form of a surface function, as one dependent variable (concentration) is attributed to two independent variables (spatial coordinate and time). The surface function is shown in Fig. 6a. A more adequate representation of how the concentration profile evolves over time could be based on the selection of only a few time coordinates. Fig. 6b demonstrates that even if the concentration profile is irregular around t=0 min, it gradually transforms into an almost perfectly sinusoidal curve; see the profile at t=2 min. The self-regulating process and the transformation that will ultimately lead to a sinusoidal function are also presented in a more conventional, two-dimensional diagram; see Fig. 6c. Videos offer a more sophisticated method to depict time dependence. Therefore, supplementary file SI2 was also prepared and portrays self-regulation animatedly.

**Fig. 7:**
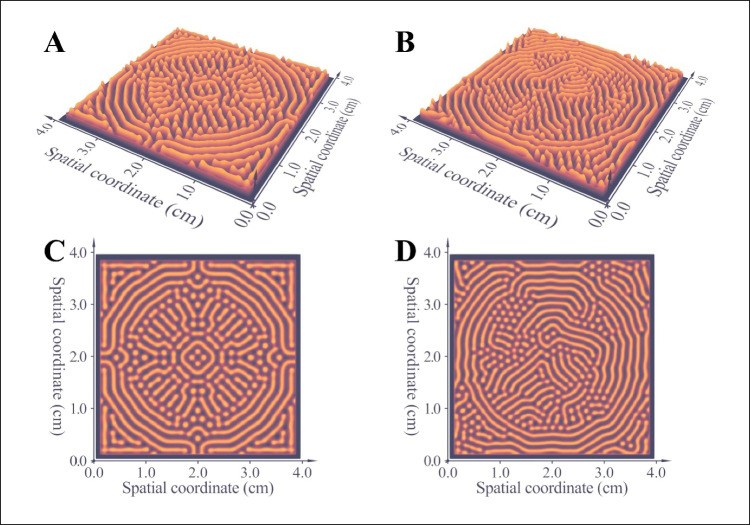
Results of modeling pattern formation along two spatial coordinates. Three-dimensional (**a**) and two-dimensional (**c**) plots of the final surface function if the simulation was started with regular initial conditions. Figures (**b**, **d**) present the results of the same simulation started with irregular initial conditions

Although the diagrams presented in Fig. 6 and the animations in supplementary video SI2 highlight the self-regulating nature of the investigated reaction-diffusion phenomena, one-dimensional simulations have little practical significance. Therefore, the equation system that consists of Eqs. 1 and 2 was solved in two dimensions as well. The results are presented in Fig. 7 and supplementary video SI3. Fig. 7a and c demonstrate the final state of a simulation that started with perfectly regular initial conditions. Conversely, the pattern observed in Fig. 7b and d are not symmetric, owing to the irregular nature of the initial conditions this simulation was carried out with. Besides the geometry of the patterns, the time dependence of the process is also similar to that observed in actual experiments. The precipitation starts at the perimeter of the sample holder and gradually progresses towards the center. This tendency is demonstrated by supplementary video file SI3.

### Utilization of self-regulation and creation of surgical meshes

**Fig. 8:**
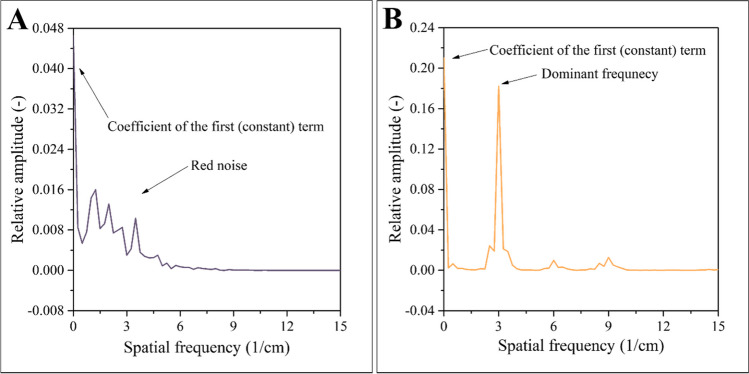
Spectrum of the concentration profile near the beginning of the experiment (**a**), and in the final (static) state (**b**). The self-regulating process functions as a bandpass filter that retains only one frequency and attenuates the rest

The most important conclusion of the simulations discussed in the previous section is that the self-regulating process leads to patterns that can be characterized by one spatial frequency – regardless of the initial conditions. We would like to draw attention to the progress displayed in Fig. 6c again: the final profile is a sinusoidal function, which can be represented by one constant wavelength. Even if the initial concentration profile was irregular, the final profile is always a regular sinusoidal of one frequency. Mathematically, self-regulation functions as a bandpass filter: it reduces the amplitude of all frequencies except for a specific one, which it allows to pass. This bandpass filter effect is graphically represented in Fig. 8. The spectrum of the concentration profile attributed to t=0 min is a red noise: it has many components; those belonging to higher frequencies have attenuated amplitudes (Fig. 8a). In comparison, the concentration profile in the final (static) state is almost a perfect harmonic function. Accordingly, its spectrum consists of only one component: one dominant peak appears at the frequency of the sinusoidal function (Fig. 8b). The amplitudes of the remaining peaks are marginal, i.e., the self-regulating process acts as a very effective bandpass filter.

**Fig. 9:**
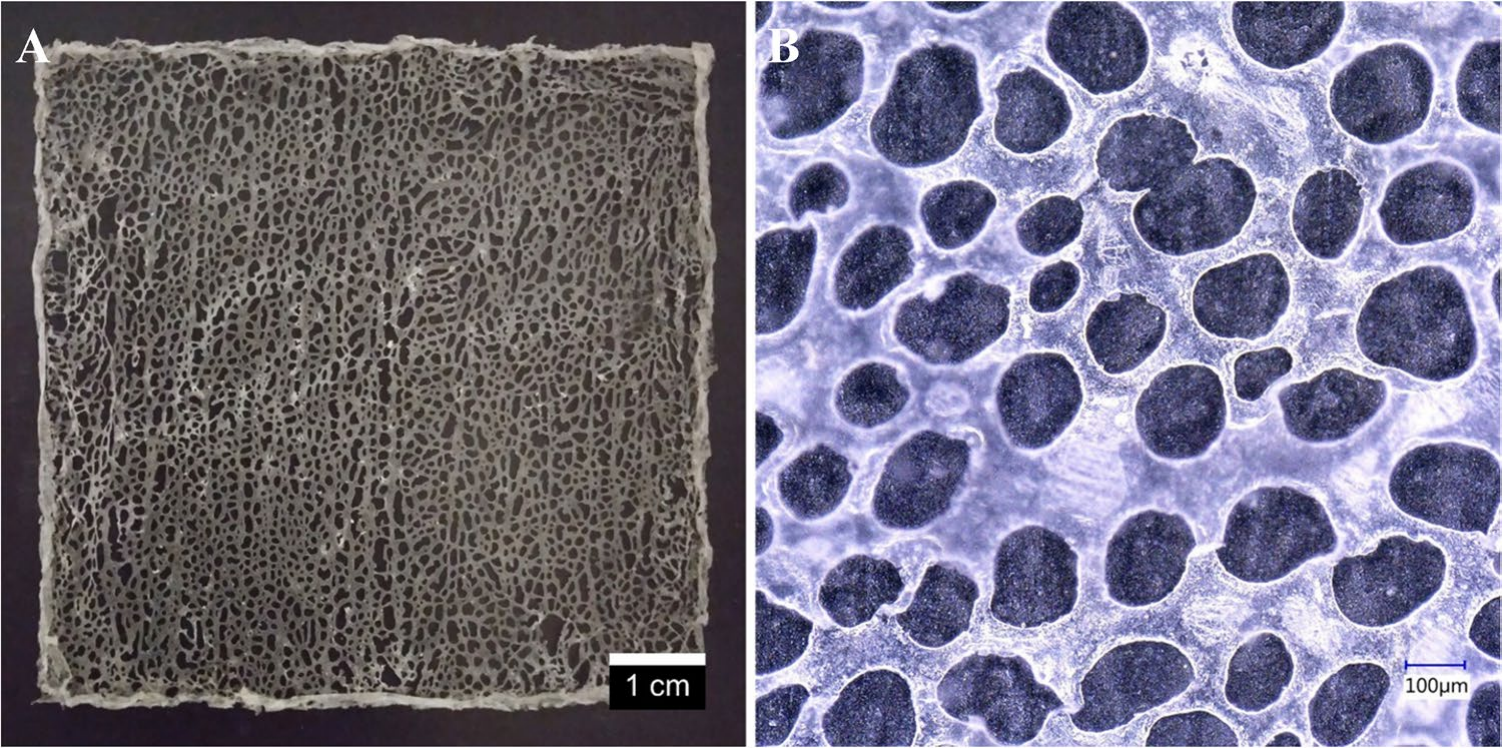
Surgical meshes with holes/pores shaped and distributed uniformly by self-regulation. Photo of a mesh with an average hole size of ~2 mm (**a**) and microscopic image of a mesh with an average pore size of ~200 μm (**b**). Note that the process yields pores of a narrow distribution of diameters, regardless of their order of magnitude

A comparison of Fig. 8b and b also reveals that the results of the modeling presented in Section 3.2 can describe empirical data very well. Consequently, we can safely assume that an experimental plan can rely on the bandpass filter effect displayed in Fig. 8. Therefore, we decided to utilize this concept in practice and created meshes with holes yielded by parallel precipitation and diffusion. Due to self-regulation and the filter effect discussed above, the holes that formed during the experiment are evenly distributed and have a narrow distribution of diameters; see Fig. 9a. Regarding this figure, we would like to point out that this kind of hole formation will be achieved if the solution of the polymer contains an additive (PEG; see the experimental section). PEG is fully biocompatible and is already used extensively under *in vivo* conditions [44–46]. Therefore, the presence of this additive will not hinder the application of surgical meshes manufactured with our method.

Another advantage of the method we propose here is that the average size of the holes can also be adjusted and tailored to the desired application. The faster the process of precipitation, the smaller the holes will be. Therefore, if a mesh with holes smaller than those displayed in Fig. 9a is required, the rate of precipitation is to be increased. A convenient and straightforward way of accelerating the precipitation is to apply less solution of the polymer onto the surface of the glassware. In this way, after the solution spreads, the thickness of the layer will be thinner, which will speed up the process. By fine-tuning the thickness of the layer of the polymer solution, we were able to achieve an average pore size as little as ~200 μm; see Fig. 9b. This figure also proves that self-regulating reaction-diffusion yields pores of a narrow distribution of diameters. While this is clearly a very beneficial feature, we must draw attention to the relationship between the uniformity of pores in woven/knitted meshes and those created with our method. Owing to the mechanical nature of pore creation during weaving or knitting, these meshes contain pores that are of the exact same size. In comparison, surgical meshes that are the result of reaction-diffusion pattern formation contain holes that are of similar, but not the exact same size.

Figure 9 also reveals that our method facilitates the creation of surgical meshes with an average hole/pore size falling in the 2 mm - 200 μm range. Several publications [24, 39, 47] mention the importance of this parameter and report that the optimal value is in the ~100 μm - few millimeters range (the exact value depends on the intended area of application [24, 39, 47]). Since the meshes created with our method cover most of this interval, they appear to be an ideal candidate for medical applications. We also would like to mention that the variability of the pore size can prove to be very helpful for surgeons looking for a mesh of an optimal pore size. While selecting a mesh that meets all requirements, surgeons often have to browse through multiple product lines. If our method is used, the product can be easily and quickly tailored to individual surgeries, eliminating the need for multiple product lines manufactured by using different methods. Furthermore, these meshes do not consist of individual fibers, unlike the ones currently available for the biomedical industry. Due to the fibrous composition and direction-dependent mechanical characteristics of contemporarily applied meshes, the surgeon must pay very close attention to their orientation during implanting [48, 49]. In contrast, the meshes shown in Fig. 9 do not consist of woven or knitted fibers. Therefore, their mechanical properties are orientation-independent, which potentially simplifies surgical procedures and reduces associated risks.

An additional advantage of this method is that it can be scaled up easily. Large-scale manufacturing is based on the continuous production of the mesh, which can be achieved as follows. Instead of a static glass plate, the solution of the polymer is to be spread on the surface of a conveyor belt coated with polytetrafluorethylene (Teflon). While being carried by the conveyor belt, the polymer precipitates and reaction-diffusion patterns (holes in this case) are formed. Then, the continuously manufactured porous mesh is separated from the conveyor belt and directed into an aqueous bath, where the additive that aids pore formation (PEG) is removed. Subsequently, the cleaned mesh is led into an online heated oven where water is removed from the surface, and the product is dried thoroughly. Lastly, the mesh is spooled/wound up into rolls that can be placed into autoclaves for sterilization. Even though the upscaled production is completely feasible, there are challenges associated with the continuous manufacturing of the mesh. Most importantly, the polymer solution must be distributed evenly on the surface of the conveyor belt; otherwise, the spatial frequency of the pattern (diameter and distance of the pores in this case) might become position-dependent.

We have seen that the method proposed in this paper offers numerous advantages. However, we also would like to point out that possible applications of this manufacturing technology are constrained by limitations, which must also be discussed. First and foremost, the method works only with copolymers of polyisobutylene. Derivatives of polyisobutylene show excellent biocompatibility and are already used *in vivo* [41–43], but if a polymer belonging to another family must be used, the feasibility of this method becomes questionable. The second limitation concerns the range of possible pore sizes. As presented in Fig. 9, the average diameter can be varied between 2 mm and 200 μm. While this range covers most of the interval that was found to be optimal for surgical meshes, there are always exceptional cases where a mesh of a pore size falling out of this range is required. Under such circumstances, this method cannot be used. Lastly, the technology is sensitive to the even distribution of the polymer solution on the surface of the carrier. When performed manually, a uniform thickness of the polymer solution can be achieved easily. However, the ultimate goal is the scaled-up production of these meshes, where evenly spreading the solution on the surface of a continuously moving and pliable conveyor belt is much more challenging.

## Conclusions

The controlled precipitation of SIBS yields reaction-diffusion patterns. The geometrical characteristics of the patterns, such as the relative amounts of dots, stripes, and holes, can be adjusted by varying the parameters of the precipitation. Pattern formation is very susceptible to minor perturbations; therefore, the same pattern cannot be created twice. In contrast, the macroscopic characteristics of the patterns, most importantly the dominant spatial frequency, are fully deterministic and easily reproducible. Spatial frequency can be measured by processing the data extracted from the photos of the patterns with Fourier transformation. The deterministic nature of dominant spatial frequency can also be modeled mathematically. The time and the spatial coordinate dependence of the concentration of each component can be described by numerically solving the partial differential equation system of the model. Modeling reveals that the process acts as a bandpass filter: the final concentration profiles will always be sinusoidal, regardless of the irregularities in the initial conditions. In practice, this means that the dominant geometric elements, e.g., dots, stripes, or holes, will be distributed uniformly. This kind of self-regulation can be utilized to create surgical meshes with evenly distributed holes that have a narrow distribution of diameters. Therefore, meshes created with our method rival the uniformity and regularity of those manufactured by weaving or knitting. In addition to meeting the industry standards for meshes currently available in the market, our technique offers further benefits. By varying the parameters of pattern formation, the average pore size of the surgical mesh can be adjusted, which facilitates the rapid tailoring of the process to the intended area of application.

## Supplementary information

ESM 1 Time lapse of the formation of reaction-diffusion patterns (animated). (MP4 22877 kb)

ESM 2 Results of the simulation carried out in one dimension (animated). (MP4 17442 kb)

ESM 3 Results of the simulation carried out in two dimensions (animated). (MP4 25683 kb)
